# From Inflammation to Tissue State: A Compartment Model for Chronic Rhinosinusitis

**DOI:** 10.1002/clt2.70207

**Published:** 2026-09-24

**Authors:** Martin Desrosiers, Francois Lavigne, Leandra Mfuna Endam, Sheherazade Jannat, Audrey Pelletier

**Affiliations:** ^1^ Centre de Recherche du Centre Hospitalier de l’Université de Montréal (CRCHUM) Université de Montréal Montréal Quebec Canada; ^2^ Department of Otolaryngology–Head and Neck Surgery Centre Hospitalier de l’Université de Montréal (CHUM) Université de Montréal Montréal Quebec Canada

**Keywords:** biologics, chronic rhinosinusitis, endoscopic sinus surgery, epithelium, extracellular matrix, genetics, nasal polyps, precision medicine, type 2 inflammation, transcriptomics

To the Editor,

The recent report by Giombi et al. provides timely ultrastructural evidence that dupilumab therapy in chronic rhinosinusitis with nasal polyposis (CRSwNP) may be associated not only with clinical improvement but also with partial restoration of mucosal architecture, including pseudostratified ciliated epithelium, epithelial junctions, and extracellular matrix organization [1]. These observations highlight an important challenge for the field: clinical response, inflammatory suppression, epithelial repair, and structural remodeling are related but non‐identical biological outcomes.

We propose that CRSwNP can be interpreted through a three‐compartment model composed of the immune system, the epithelium/barrier unit, and the extracellular matrix (ECM)/remodeling compartment (Figure 1). Each compartment contains distinct dysregulated pathways, but each also influences the others. This framework may help clinicians and investigators interpret why therapies with comparable clinical benefit may differ in mechanism, timing, durability, and depth of tissue normalization.

**FIGURE 1:**
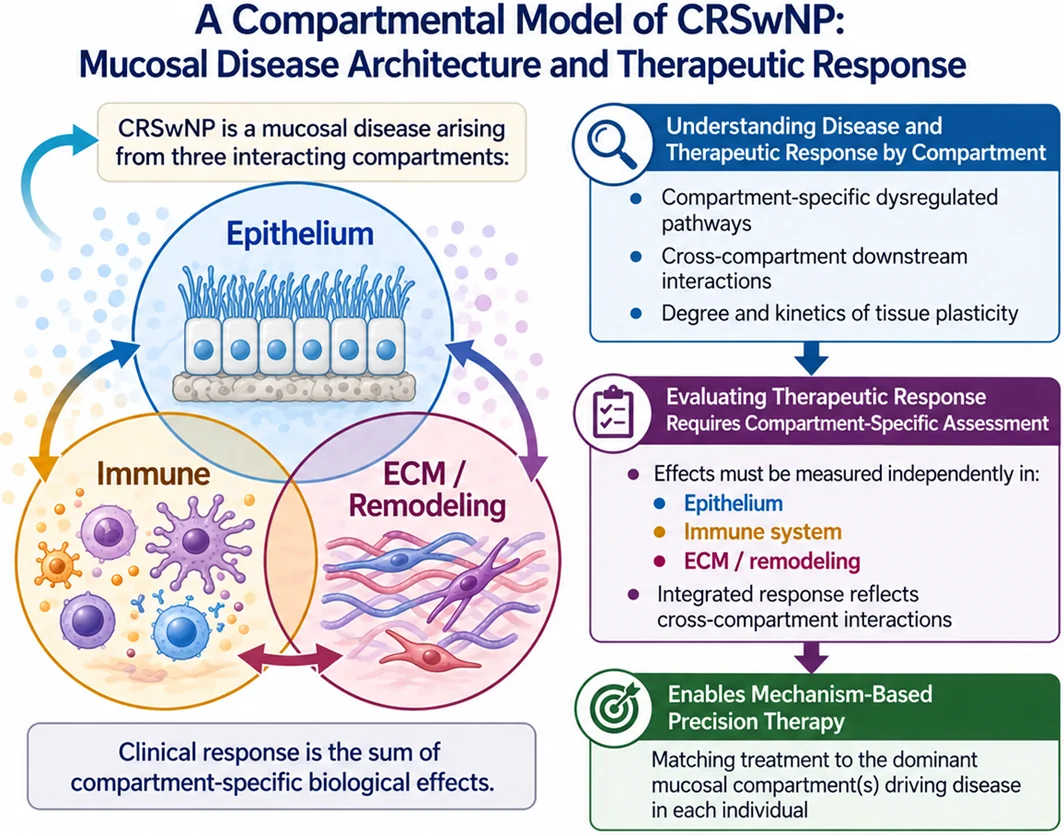
A compartmental model of CRSwNP. CRSwNP is conceptualized as a mucosal disease arising from interactions among the epithelium/barrier, immune, and ECM/remodeling compartments. Each compartment may contain distinct dysregulated pathways while influencing the others through cross‐compartment signaling. Clinical response is proposed to reflect the integrated sum of compartment‐specific biological effects. This framework may help compare therapeutic mechanisms, contextualize susceptibility genetics, and support mechanism‐based treatment selection.

The immune compartment is dominated by type 2 inflammation in many CRSwNP populations, with IL‐4, IL‐5, IL‐13 signaling, eosinophilic inflammation, and innate and adaptive immune activation providing the rationale for current biologic therapies [2, 3]. However, type 1 programs, including interferon‐ and TNF‐associated signaling, may also contribute to disease heterogeneity and epithelial stress. In this model, inflammatory pathways are positioned primarily within the immune compartment, whereas their consequences extend to epithelial differentiation, barrier function, and tissue remodeling.

The epithelial compartment should be viewed as an active disease driver rather than a passive surface injured by inflammation. In CRSwNP, epithelial abnormalities include impaired barrier integrity, altered differentiation, defective mucociliary clearance, and production of epithelial‐derived cytokines such as IL‐25, IL‐33, and thymic stromal lymphopoietin [3, 4]. The ciliated epithelium therefore represents a functional readout of mucosal health. The ultrastructural finding of ciliated epithelial regrowth during dupilumab therapy is particularly relevant because it suggests that immune modulation can permit downstream epithelial maturation in selected patients [1].

The ECM/remodeling compartment captures the structural consequences of chronic disease, including edema, fibrin deposition, altered coagulation and fibrinolysis, collagen remodeling, and stromal reorganization [5]. This compartment may explain why control of inflammation does not always produce complete normalization of tissue structure, and why surgery remains necessary in many patients with obstructive, remodeled, or poorly accessible disease.

When we interpret the ultrastructural findings of *Giombi* et al. using this three‐compartment framework, we can observe changes in each of the three compartments. Following 12 months of dupilumab, reduced tissue inflammatory infiltration in the immune compartment is associated with downstream epithelial and barrier restoration, including pseudostratified ciliated epithelial regrowth, renewed ciliary ultrastructure, and partial junctional reassembly. Parallel ECM remodeling includes improved vascular organization and collagen restoration.

This compartmental framework also clarifies therapeutic differences. Dupilumab and mepolizumab act primarily through immune modulation, with downstream epithelial and remodeling effects that may vary by a baseline tissue state [6, 7]. Endoscopic sinus surgery, in contrast, directly modifies the epithelial and ECM compartments by removing obstructive tissue, restoring ventilation, improving topical access, and creating conditions for repair; its immunologic effects are likely secondary to alteration of the local mucosal environment [8]. These interventions should therefore be considered compartment‐selective and potentially complementary rather than interchangeable.

Importantly, this model cautions against equating symptomatic improvement with complete remission. Giombi et al. observed clinical improvement across patients, but persistence of ultrastructural abnormalities in a subset [1]. This dissociation supports a multidimensional view of response: one patient may achieve immune control without complete epithelial repair, whereas another may regenerate ciliated epithelium yet retain residual ECM or inflammatory memory. Such distinctions may help explain recurrence, incomplete response, and the need for combined or sequential therapy.

A further advantage of the compartment model is that it provides a contextual scaffold for interpreting susceptibility genetics. Prior genetic studies, including candidate‐gene and genome‐wide association approaches, suggest that CRS has a heritable component, but assigning biological meaning to candidate variants remains difficult when disease is treated as a single entity [9]. Mapping emerging variants to immune, epithelial/barrier, or ECM/remodeling compartments may help estimate their likely functional consequences and connect genetic risk to mechanistic hypotheses. Although sequencing‐based CRS diagnosis is not yet routine, if it becomes clinically available, this structure could help prioritize variants of significance and support automated interpretation within biologically meaningful compartments.

In summary, a compartment‐based model offers a practical translational bridge between endotype, tissue state, therapeutic mechanism, and susceptibility genetics in CRSwNP. By evaluating immune, epithelial/barrier, and ECM/remodeling responses separately, the field may move from asking whether a treatment works toward understanding where it acts, how deeply it repairs mucosa, which genetic risks may shape disease, and in whom therapy is most likely to produce durable benefit.

## Author Contributions

Martin Desrosiers conceived the proposed compartment model and prepared the initial manuscript draft. François Lavigne, Leandra Mfuna Endam, Sheherazade Jannat, and Audrey Pelletier contributed to the development and refinement of the conceptual framework and critically revised the manuscript for important intellectual content. All authors reviewed and approved the final version of the manuscript and accept responsibility for its content.

## Funding

The authors have nothing to report.

## Ethics Statement

The authors have nothing to report.

## Conflicts of Interest

Desrosiers: Clinical trial funding: Sanofi, Regeneron, GSK. Advisory boards: Sanofi, Regeneron, GSK, AstraZeneca. Equity stakeholder: Probionase Therapies Inc. Francois Lavigne: No conflicts to declare. Leandra Mfuna Endam: No conflicts to declare. heherazade Jannat: No conflicts to declare. Audrey Pelletier: No conflicts to declare.

## Data Availability

No new data were generated or analyzed in support of this article. This article presents a conceptual framework that integrates previously published observations and hypotheses to propose a new approach to understanding and interpreting the pathophysiology of chronic rhinosinusitis with nasal polyps. Data sharing is therefore not applicable.
